# Analysis and Characterization of Glutathione Peroxidases in an Environmental Microbiome and Isolated Bacterial Microorganisms

**DOI:** 10.4014/jmb.2209.09006

**Published:** 2023-01-20

**Authors:** Yun-Juan Bao, Qi Zhou, Xuejing Yu, Xiaolan Yu, Francis J. Castellino

**Affiliations:** 1 State Key Laboratory of Biocatalysis and Enzyme Engineering, Hubei Key Laboratory of Industrial Biotechnology, School of Life Sciences, Hubei University, Wuhan 430062, P.R. China; 2 W. M. Keck Center for Transgene Research, University of Notre Dame, Notre Dame, Indiana 46556, USA; 3 Department of Chemistry and Biochemistry, University of Notre Dame, Notre Dame, Indiana 46556, USA

**Keywords:** Microbial genomes, phylogeny, glutathione peroxidase, peroxiredoxin, structure

## Abstract

Glutathione peroxidases (Gpx) are a group of antioxidant enzymes that protect cells or tissues against damage from reactive oxygen species (ROS). The Gpx proteins identified in mammals exhibit high catalytic activity toward glutathione (GSH). In contrast, a variety of non-mammalian Gpx proteins from diverse organisms, including fungi, plants, insects, and rodent parasites, show specificity for thioredoxin (TRX) rather than GSH and are designated as TRX-dependent peroxiredoxins. However, the study of the properties of Gpx in the environmental microbiome or isolated bacteria is limited. In this study, we analyzed the Gpx sequences, identified the characteristics of sequences and structures, and found that the environmental microbiome Gpx proteins should be classified as TRX-dependent, Gpx-like peroxiredoxins. This classification is based on the following three items of evidence: i) the conservation of the peroxidatic Cys residue; ii) the existence and conservation of the resolving Cys residue that forms the disulfide bond with the peroxidatic cysteine; and iii) the absence of dimeric and tetrameric interface domains. The conservation/divergence pattern of all known bacterial Gpx-like proteins in public databases shows that they share common characteristics with that from the environmental microbiome and are also TRX-dependent. Moreover, phylogenetic analysis shows that the bacterial Gpx-like proteins exhibit a star-like radiating phylogenetic structure forming a highly diverse genetic pool of TRX-dependent, Gpx-like peroxidases.

## Introduction

Reactive oxygen species (ROS), such as free radicals and peroxides, are produced in the cells of organisms under oxidative stress, causing the cells extensive damage. This demonstrates that organisms have developed diverse antioxidant mechanisms for protection against cellular damage. The antioxidant system includes the small molecular nonenzymatic antioxidants (*i.e.*, vitamin C, vitamin E, and *et al*.) to repair the damage, and the antioxidant enzymes to metabolize the ROS. The antioxidant enzymes include superoxide dismutase (E.C. 1.15.1.1), catalase (E.C. 1.11.1.6), peroxiredoxin (E.C. 1.11.1.7), and glutathione peroxidase (E.C. 1.11.1.9). Peroxiredoxin (Prx) and glutathione peroxidase (Gpx) are the mostly widely studied due to their primary role in protecting cells from oxidative damage caused by ROS.

Peroxiredoxins include a large family of thiol-specific antioxidant peroxidases usually using TRX as the reducing agent. This family of proteins is ubiquitous in almost all domains of life and is generally divided into four subtypes, specifically, typical 2-Cys, atypical 2-Cys, 1-Cys, and PrxQ [1, 2]. All the subtypes share a conserved Cysteine (Cys) residue at the N-terminus, designated as peroxidatic Cys, which facilitates a nucleophilic attack on the peroxides to generate a sulfenic acid. The four subtypes generally differ in their quaternary structures and the location of the second Cys, termed resolving Cys, which eliminates the oxidized Cys by forming a disulfide bond with the peroxidatic Cys. The typical 2-Cys group has a resolving Cys at the C-terminus which forms an intermolecular disulfide bond with a second subunit. The atypical 2-Cys group also contains a resolving Cys, but the Cys has a varied location on the protein and usually forms an intramolecular disulfide bond with the peroxidatic Cys within the same subunit. The 1-Cys type only contains a peroxidatic Cys and forms a disulfide bond with alternative thiols. Lastly, the PrxQ members mostly contain both conserved peroxidatic Cys and resolving Cys on the same helix and the two Cys residues form an intramolecular disulfide bond [1].

Glutathione preoxidases catalyze the reduction of hydrogen peroxide using GSH as the reducing agent. Although Gpx has been discovered in almost all domains of life, these enzymes have been primarily studied in mammals, wherein eight isoforms, Gpx1-Gpx8, have been identified [3-6]. Most of the identified mammalian Gpx are selenoproteins and employ a selenocysteine (SeCys) in place of Cys at the catalytic site. However, mammalian Gpx5, Gpx7, and Gpx8 have unknown electron donors or low Gpx activity. Those SeCys-based mammalian Gpx proteins have been named canonical Gpx and share common primary sequences and tertiary structures. While the SeCys-based canonical Gpx proteins share a similar thioredoxin fold with the Prx members in their tertiary structure, they are phylogenetically distant from the Prx family with sequence similarities lower than 20% [7]. They also harbor significantly different signatures from Prx. First, the SeCys is responsible for the redox cycle and a resolving Cys is absent. Secondly, the canonical mammalian Gpx proteins contain a functional helix/dimeric loop with the motif “PGGG” that contributes to dimeric interactions and a variable oligomerization loop at the C-terminus that mediates contacts between two dimers [7].

On the other hand, the non-mammalian Gpx orthologs normally have a Cys residue at the catalytic site and use TRX rather than GSH as the reducing agent. Recently, numerous orthologs of Gpx were identified in a variety of non-mammalian organisms, such as fungi [8, 9], plant [10, 11], insects [12], and rodent parasites [13-15]. Those non-mammalian Gpx orthologs exhibit significant sequence and structural similarities with the canonical mammalian Gpx, but were shown to have a higher preference for TRX over GSH, as the electron donor and should be functionally classified as Prx. Physiochemical study of the Gpx-like proteins in several non-mammalian organisms, such as Plasmodium [15], *Drosophila* [12], *Chlorella* [11], and *Trichoderma* [9], has confirmed the catalytic preference for TRX over GSH. The biochemical mechanisms of the redox interaction between Gpx and TRX from *Arabidopsis* were also investigated [16]. Indeed, it has been proposed that the majority of the non-mammalian Gpx-like peroxidases have substrate specificity for TRX and are more ancient than the canonical mammalian Gpx proteins, which use GSH as the reductant [17, 18].

The crystal structures of several Gpx-like proteins from non-mammalian organisms, such as yeast [8], poplar [10], and *Drosophila* [12] were derived to investigate the genetic and structural characteristics of the redox specificity. On one hand, the studies showed that the TRX-dependent Gpx-like proteins share common structural properties with the canonical Gpx proteins, viz.,: i) both form the typical thioredoxin-fold with four strands forming internal β sheets surrounded by four α-helices which are then interconnected by several loops; (ii) both form a tetrad redox center comprising four residues, Cys (or SeCys), Gln, Trp, and Asn, where the Cys or SeCys residue is the peroxidatic residue and facilitates the nucleophilic attack on the hydroperoxides by forming hydrogen bonds with Gln and Trp [19]. On the other hand, the TRX-dependent, Gpx-like proteins exhibit significant differences from the canonical Gpx proteins: (i) a highly conserved peroxidatic Cys has been identified in TRX-dependent, Gpx-like proteins, while SeCys was found in the canonical Gpx proteins; (ii) another so-called “resolving Cys” is present in TRX-dependent, Gpx-like proteins but not the canonical Gpx, which is involved in the catalytic cycle by forming a disulfide bond with the peroxidatic Cys. The resolving Cys was considered to be required for the Gpx orthologs to use TRX as the electron donor through formation of an intramolecular disulfide bond with the peroxidatic Cys [17]; (iii) the oligomeriazation domain for formation of the multimeric structure is lacking in TRX-dependent, Gpx-like proteins, which is typical of the canonical Gpx proteins. Notably, those characteristics distinguish TRX-dependent, Gpx-like proteins from those of canonical Gpx and align with those of some PRX family proteins, *e.g.*, the PrxQ subtype, supporting the common catalytic substrate and mechanism with this family [1].

The presence of the Gpx-like proteins in bacteria has been regarded as widespread and even ancient [20]. However, the details of the sequences, structures, and phylogeny of this class of proteins in bacteria have not been sufficiently investigated and the identification of the proteins in the environmental microbiome has not been reported. Environmental microbiomes are a rich source for discovering novel genes. In particular, the microbiome from soils has the potential to evolve a rich pool of antioxidant proteins due to the oxidative stresses in the soil niche.

In the present study, we leverage this potential of the soil microbiome by mining these data from our in-house dataset (PRJNA237577 and SRP036853) and the NCBI environmental sequence dataset (env_nr) [21]. We investigated the characteristics of Gpx-like proteins from environmental microbiome metagenomes and we isolated bacterial species in terms of sequences, structures, and phylogeny. Furthermore, we provide evidence that most of the Gpx-like proteins encoded by the environmental microbiome and isolated bacterial species should be classified as TRX-dependent, Gpx-like peroxidases with TRX as the reducing agent.

## Materials and Methods

### Structure Data of Known Gpx-Like Proteins

The Gpx-like protein sequences with known biochemical properties or structure information were derived from UniProt [22] or the PDB database (www.rcsb.org), which includes yeast Gpx3 [8] (PDB ID: 3CMI), populous Gpx5 [10] (PDB ID: 2P5Q), *Drosophila* DmGPx [12] (UniProt accession no. AAF47761), Schistosome Gpx4 [13](PDB ID: 2V1M), and eight human Gpx (Gpx1 with PDB ID: 2F8A, Gpx2 with PDB ID: 2HE3, Gpx3 with PDB ID: 2R37, Gpx4 with PDB ID: 2OBI, Gpx5 with UniProt accession no. O75715, Gpx6 with UniProt accession no. P59796, Gpx7 with PDB ID: 2P31, and Gpx8 with PDB ID: 3CYN). The multiple sequence alignment was performed using Clustal Omega (version 1.2.2) [23] and adjusted by manual curation. The graphical presentation of the final alignment was prepared with Espript (version 3.0) [24]. The sequences of the above Gpx-like proteins were assembled as a set of known Gpx or Gpx-like proteins.

### Gpx-Like Protein Sequences from Environmental Microbiome and Isolated Bacterial Species

The microbiome Gpx-like proteins were obtained from the metagenomes of the data depository SAMN02630628 and NCBI env_nr database (last accessed on Oct. 6, 2021) (www.ncbi.nih.gov). The metagenomic sequences from the depository SAMN02630628 were assembled using velvet (version 1.0.18) [25] and the genes were predicted using MetaGeneMark (version 3.25) [26]. The Gpx-like proteins from the environmental microbiome datasets were extracted based on functional annotation of “glutathione peroxidase” or by a BLAST homology search against a set of previously prepared Gpx proteins (see the previous section in Materials and Methods).

The bacterial Gpx-like proteins were collected from the UniProt database using two methods [22]: (i) by advanced search with the keyword “glutathione peroxidase” in “Gene Name” and “bacterium” in “Taxonomy” on the web at www.uniprot.org; (ii) by sequence comparison using BLAST against a set of previously prepared Gpx proteins. The protein entries derived from the two methods were combined and de-duplicated. The extracted proteins were processed by discarding those sequences that were too short or too long and trimming the divergent N-terminus and C-terminus in the multiple sequence alignment. The non-redundant protein set was derived by performing cd-hit on the processed proteins [27]. The multiple sequence alignment of the Gpx-like proteins was performed using Clustal Omega (version 1.2.2) [23] and the conservation pattern was identified using ConSurf (version 3) [28].

### Structural Modeling

The three-dimensional structure of MtGpx0 was modeled using the crystal structure of Schistosome Gpx4 2V1M as the template (with similarity ~ 43%) on the Phyre server (version 2.0) [29]. The structure model was also predicted using AlphaFold2 [30]. All the structures were presented and analyzed with PyMOL (version 2.5.1)[31]. The model covers residues 28-186 of MtGpx0. A robust structure is lacking at the extreme N-terminus due to the high diversity of the sequences at this region which consequently yielded these diverse structures.

### Phylogenetic Analysis

The phylogenetic tree was constructed for the multiple aligned sequences using MEGA (version 6) [32] with bootstrap of greater than 1000 using the neighbor-joining method. The phylogenetic network for the same set of Gpx-like proteins was generated by SplitsTree (version 4.15.1) using the neighbor-net method [33]. To reduce the influence of alignment gaps on tree building, the large gap regions, such as the oligomerization domain in the multiple aligned sequences, were removed.

## Results

### Comparison of the Primary Sequences of Gpx-Like Proteins from Microbiome Metagenomes with the Gpx or Gpx-Like Proteins from Non-Microbial Species

A total of 1,319 Gpx-like proteins were identified from the environmental microbiome metagenomes in the combined NCBI env_nr dataset and our in-house dataset (see Materials and Methods). Using this set of proteins, we created a non-redundant set of 392 proteins (averaged to 186 amino acids in length) by merging the proteins with pair-wise similarities greater than 80%. Multiple sequence alignment of the 392 proteins exhibits a well-conserved pattern (Fig. S1). Construction of their phylogenetic tree shows that many proteins are closely clustered (Fig. S2), and therefore we further selected 26 representative proteins, which are able to cover the major branches and are distant from each other (the similarities averaged to 45.7% and range between 40-65%) (Fig. S3A).

**Fig. 1:**
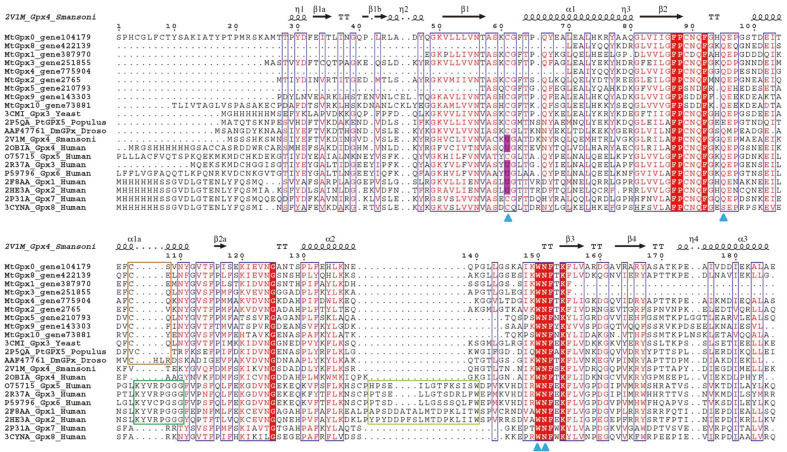
Multiple sequence alignments of Gpx or Gpx-like proteins. The Gpx-like proteins from current study include MtGpx0, MtGpx1, MtGpx2, MtGpx3, MtGpx4, MtGpx5, MtGpx8, MtGpx9, and MtGpx10. Those with known structural information include yeast Gpx3 3CMI, populous Gpx5 2P5QA, *Drosophila melanogaster* DmGPx AAF47761, *Schistosome mansoni* Gpx4 2V1M, human Gpx4 2OBIA, human Gpx5 O75715, human Gpx3 2R37A, human Gpx6 P59796, human Gpx1 2F8AA, human Gpx2 2HE3A, human Gpx7 2P31A, and human Gpx8 3CYNA. The secondary structure was obtained from *Schistosome mansoni* Gpx4 and displayed on the top of the alignment. The α-helices and 3_10_-helices are represented by coils and labeled by α and η respectively. The β-strands are represented by arrows and labeled by β. The β turns are labeled by TT. The identical residues in the same columns are shaded in red and displayed in white letters, while homologous residues are displayed in red letters and framed in blue boxes. The catalytic residues (Cys61 or SeCys61, Gln95, Trp150, and Asn151) are indicated with blue triangles at the bottom of the alignment. The SeCys residues (IUPAC letter U) were shaded with pink. The block containing resolving Cys, *i.e.*, “Cys block” is framed with orange. The regions for dimer interface loop (the functional helix) and tetramer interface loop (the oligomerization loop) in the five human Gpx proteins are framed in green and light green, respectively.

To further assess the similarity between the environmental microbiome-encoded Gpx-like proteins and the non-microbial Gpx-like proteins, we performed multiple sequence comparison between nine representatives of the 26 microbiome-encoded Gpx-like sequences and those encoded by yeast, poplar, *Drosophila*, *Schistosomes* and humans (Fig. 1). As a result, the microbiome-encoded Gpx-like sequences were shown to be highly similar to the known non-mammalian Gpx-like Prx proteins (yeast Hyr1, poplar PtGpx5, *Drosophila* DmGpx) or phospholipid peroxidases (*Schistosomes* SmGpx and human Gpx4) with average similarities of ~42% compared to the ~30%with the canonical human Gpx proteins (Gpx1-Gpx3 and Gpx5-8). The relationship is also supported by their phylogenetic structure, where the microbiome-encoded Gpx-like proteins are clustered together with yeast Hyr1, poplar PtGpx5, *Drosophila* DmGpx, *Schistosomes mansoni* SmGpx, and human Gpx4 (Fig. S3B). There is no detectable sequence similarity between the microbiome Gpx-like proteins with the canonical bacterial Prx proteins (<20%). This indicates that the microbiome-encoded Gpx-like proteins are closely related with the Gpx-like Prx proteins at the primary sequence level.

### The Gpx-Like Proteins from Microbiome Metagenomes Exhibit Genetic Properties Analogous to TRX-Dependent Prx

We performed comparative analysis of the primary sequences and secondary structures of the Gpx-like proteins from the environmental microbiome dataset and non-microbial species to examine the characteristics of the environmental microbiome-encoded Gpx-like proteins. We found that the four active site tetrad residues (SeCys61 or Cys61, Gln95, Trp150, and Asn151, blue triangles in Fig. 1) are conserved among the compared sequences, except that the peroxidatic Cys is replaced by SeCys in mammalian Gpx (except in Gpx5, Gpx7, and Gpx8) (encoded by the IUPAC letter U, shaded in pink in Fig. 1) [34]. A second Cys residue, Cys107, was found in a region called “Cys block” on the α1a helix in the Gpx-like sequences from the environmental microbiome metagenomes and the other three organisms (yeast, poplar, and *Drosophila*) (framed in orange), but not in the canonical human Gpx proteins. This second Cys residue in the “Cys block” was identified as the “resolving Cys” by forming a disulfide bridge with the peroxidatic Cys in the non-mammalian Gpx [8, 10, 12]. The disulfide bridge was shown to be essential for regeneration of the redox state of Prx in the catalytic cycle [12] and has been proposed to be specific for reducing the TRX rather than GSH [35]. Actually, we found that the resolving Cys was highly conserved among the Gpx-like proteins from the environmental microbiome dataset (>99%), thus indicating the indispensable role of this Cys in the catalysis of the environmental microbiome (Fig. S1). The high prevalence of the two Cys residues in the Gpx-like proteins is analogous to that in the 2-Cys Prx enzymes, except that the location of the resolving Cys in the latter group is variable among different Prx subtypes [1, 36].

Another notable feature of the microbiome-encoded Gpx-like proteins is the lack of the dimer loop/functional helix (framed in green in Fig. 1) and the tetramer loop/oligomerization loop (framed in light green in Fig. 1) in comparison with the canonical human Gpx, where the two loop domains are responsible for subunit interface interaction. This suggests that the microbiome-encoded Gpx-like proteins are unable to form oligomeric quaternary structures (see next sections). The absence of the multimeric domains was also observed in other TRX-dependent Gpx-like proteins, such as the monomeric *Drosophila* DmGpx and yeast Hyr1, in analogy to the Prx subtype PrxQ [1]. The exception is the dimeric poplar PtGPX5, where its dimerization is not induced from the typical dimer loop domain but from sporadic hydrophobic or polar residues [10].

### The Gpx-Like Proteins from Microbiome Metagenomes Share a Core Tertiary Structure with Gpx and Prx Family Proteins

**Fig. 2:**
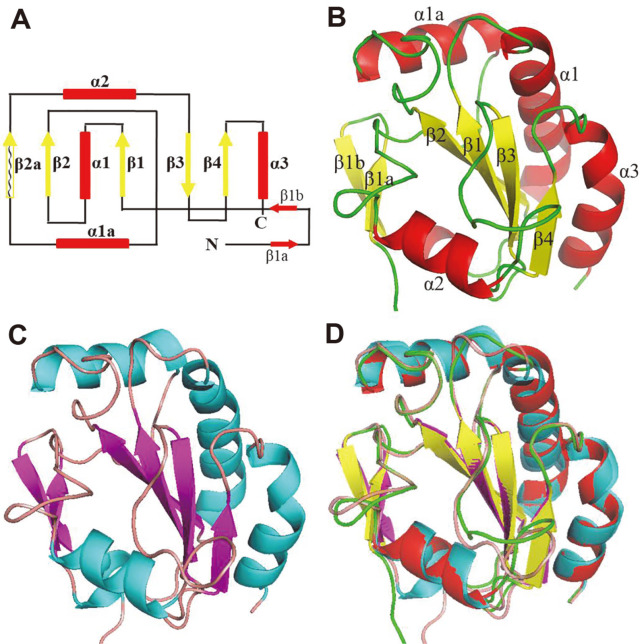
The topological diagram and the ribbon representation of the modeled structure of MtGpx0. (**A**) The topological diagram of MtGpx0 from N-termini to C-termini with red boxes for α-helices and yellow arrows for β-sheets. The wave-shaped curve enclosed in the box representing β2a indicates the low-confidence modeling for this fragment. (**B**) The ribbon representation of the modeled structure of MtGpx0 using Phyre2 with *Schistosome mansoni* SmGpx as the template. (**C**) The ribbon representation of the modeled structure of MtGpx0 predicted with AlphaFold2. (**D**) The superposition of the structural models using Phyre2 and AlphaFold2. The β2a sheet was shown as loop due to its low confidence from the modeling.

To investigate the functional implications of the microbiome-encoded Gpx-like proteins, we studied their structural properties. Considering that the pair-wise similarities between the microbiome-encoded Gpx-like proteins are high (40-65%) and the key functional domains are conserved, we used the longest sequence (MtGpx0, 186 residues) as a representative basis for building the structural model using homology modeling with Phyre2 [29] and deep learning prediction with AlphaFold2 [30] (Figs. 2A-2C). The structures from the two methods are highly consistent with the RMSD of 0.59 Å (Fig. 2D). The modeling showed that the protein MtGpx0 forms the thioredoxin-fold typical of both Gpx and Prx as the four internal β-strands form the central β sheets flanked by three α-helices [37]. The fold comprises the N-terminal motif β1α1β2 and the C-terminal motif β3β4α3 connected by the helices α1a and α2 (Fig. 2A). The modeled structure also contains the variable extension or insertion fragments typical of Gpx and Prx family proteins, including two additional β-strands at the N-terminus, *i.e.*, β1a and β1b, folding into a β hairpin, and an extra α helix α1a inserted at the proximity of the C-terminus [38].

### The Gpx-Like Proteins from Environmental Microbiome Metagenomes Share Functional Motifs with the Trx-Dependent Prx Proteins

**Fig. 3:**
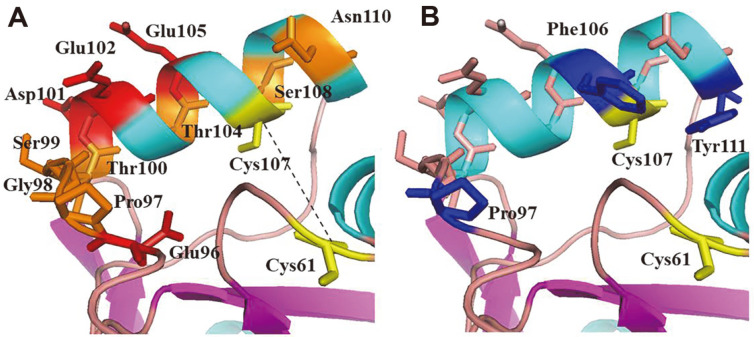
The surrounding residues of the disulfide bond. (**A**) The surrounding environment of the disulfide bond is rich in negatively charged residues, *i.e.*, Glu96, Asp101, Glu102, and Glu105 (in red) or turn-favoring residues, *i.e.*, Pro97, Gly98, Ser99, Thr100, Thr104, Ser108, and Asn110 (in orange). (**B**) The Trx-binding specific residues are located around the disulfide bond, including Pro97, Phe106, and Tyr111 (in blue).

In the modeled structure of MtGpx0, the resolving Cys107 on the helix α1a faces the peroxidatic Cys61 in the loop preceding α1. This conformation enables the two Cys to form the intramolecular disulfide bond. The formation of the disulfide bond was shown to be required for reacting with TRX [7]. The bond distance in the simulated model is estimated to be 14.3 Å, which is out of the range of the valence bond interaction. The large distance corroborates the previous observation that structural rearrangements on the Cys-containing fragments are necessary to form the disulfide bond [37]. We observed that the helix α1a and the surrounding loop are rich in residues with negatively charged residues (Glu96, Asp101, Glu102, and Glu105), or residues favoring turn-like structures (Pro97, Gly98, Ser99, Thr100, Thr104, Ser108, and Asn110) (Fig. 3A). The negative charge could cause the instability of the helix and lead to unwinding of the helix α1a, as demonstrated in the oxidized form of TRX-dependent, Gpx-like protein from poplar [10]. Therefore, those residues confer flexibility to α1a and the surrounding loop, facilitating the formation of the disulfide bond. The resolving Cys is absent in canonical human Gpx proteins.

The TRX-binding specific residues are localized around the extra helix, α1a, including Pro97, Phe106, and Tyr111 (Figs. 1 and 3B). These residues are highly conserved among the microbiome-encoded Gpx-like proteins (Figs. 1 and S1) and have been shown to be involved in TRX recognition in plant Gpx [10]. In contrast, the residues specific for GSH binding (Arg57, Arg185, and Met147 in bovine Gpx [3]) are absent in the microbiome-encoded Gpx-like proteins, further supporting the functional relationship of the Gpx-like proteins with TRX-dependent Prx.

**Fig. 4:**
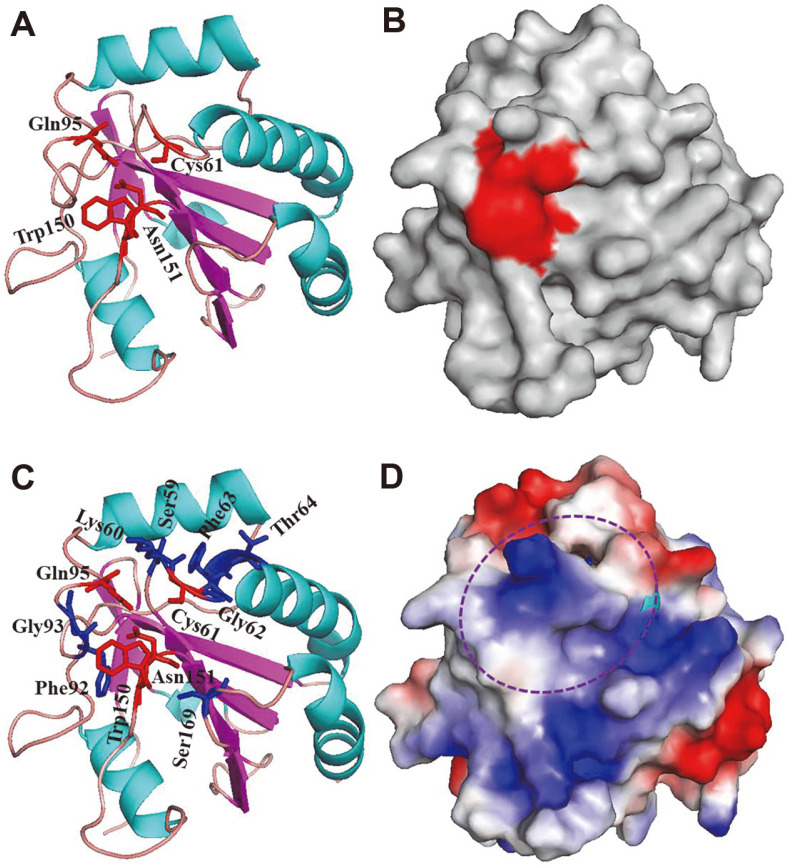
The active site cleft and the surface charge distribution. (**A**) The four active site residues are shown as red sticks. (**B**) The cleft formed by the active residues on the surface is highlighted in red. (**C**) The surrounding residues of the active site cleft are shown as blue sticks, including the non-charged Ser59, Gly62, Phe63, Thr64, Phe92, Gly93, Ser169 and positivecharged Lys60. (**D**) The surface charge distribution is colored from negative (red) to positive (blue). The residues in (**C**) induce mixed neutral and positive-charged surface (framed in purple circle).

The tetrad active site residues (Cys61, Gln95, Trp150, and Asn151) form a cleft on the structure surface with a relative solvent-accessible area of 52.7%, making it accessible to the solvent (Figs. 4A and 4B). This ratio is comparable to the 44% for *Schistosomes* SmGpx and 34% for poplar PtGPX5. The active tetrad was surrounded by several non-charged residues, *i.e.*, Ser59, Gly62, Phe63, Thr64, Phe92, Gly93, and Ser169, and one positively charged residue, Lys60 (Fig. 4C). The distribution of the surrounding residues leads to a mixed, non-charged and weak positively charged surface (Fig. 4D), in contrast to the uniformly distributed surface charges in SeCys-containing mammalian Gpx proteins in the active site cleft [10].

**Fig. 5:**
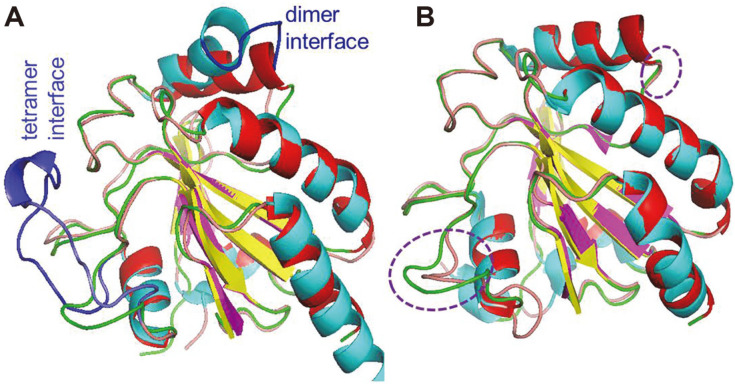
Structural alignment of MtGpx0 with the tetrameric human Gpx3 in (A) and monomeric human Gpx4 in (B). MtGpx0 is deviated from the subunit of tetrameric Gpx3 in the dimer interface (the so-called functional helix containing the peroxidatic Cys) and tetramer interface domain (the so-called oligomerization loop) of Gpx3 (highlighted in blue in A), but shows higher coincidence with the monomeric human Gpx4 in the two regions (highlighted in purple circles).

To explore the oligomerization state of MtGpx0, the modeled structure was aligned to the subunit of tetrameric human Gpx3 and monomeric human Gpx4 (Fig. 5). MtGpx0 deviates from the subunit of tetrameric Gpx3 (with an RMSD of 0.737), more so than the monomeric Gpx4 (with an RMSD of 0.394). The difference between MtGpx0 and human Gpx3 is remarkable at the dimer interface and tetramer interface of Gpx3 and is mechanistically induced by the absence of the two interface domains in MtGpx0 (Figs. 5A and 1). Instead, MtGpx0 is well superimposed on the monomeric human Gpx4 in the two regions (Fig. 5B). The structural comparison at the oligomeric interfaces strongly indicates the monomeric state of MtGpx0. Until now, all the reported TRX-dependent, Gpx-like proteins are monomers, except poplar PtGPX5, of which the dimerization is induced from alternative mechanisms other than oligomeric interface domains [10].

Taken together, the structural properties presented here for MtGpx0, *i.e.*, the conformation of the two Cys residues, the substrate binding sites, the surface charge distribution, and the oligomeric state, clearly point to an explanation as to the functional homology of MtGpx0 with the TRX-dependent Prx in spite of the high sequence similarity with the canonical Gpx proteins.

### Patterns of Sequence Conservation among All Bacterial Gpx-Like Proteins

Using sequence alignment and structural modeling, we have shown that Gpx-like proteins from environmental microbiome metagenomes harbor the key functional motifs required for the TRX-dependent catalysis. To determine whether the functional motifs related with the TRX-dependence could be extended to other bacterial Gpx-like proteins, we collected the known bacterial proteins homologous to Gpx-like proteins from the UniProt database and performed preprocessing to improve the sequence quality (see Materials and Methods) [22]. The final processed sequence set contains 1,997 entries with comparable lengths and identifiable homologies. The 1,997 protein entries were then used for multiple sequence alignment and conservation pattern profiling.

Based on the primary sequence and secondary structure alignments, we established the conservation pattern as shown in gradient colors (Figs. S4A and S5). The proteins have pair-wise similarities of 35-55%, averaging to 45%(Fig. S4B). We observed that the conservation pattern of the bacterial Gpx-like proteins is consistent with that of the environmental microbiome-encoded Gpx-like proteins, suggesting their functional homology (Figs. S4A and S1). From the sequence alignment profile, four conserved domains were obtained (Table 1). Overall, the conserved domains represent 53% of the whole protein length involving 99 residues. The four conserved domains contain the residues essential for TRX-dependent catalysis: the four active site residues (Cys, Gln, Trp, and Asn, underlined), the resolving Cys residue (double underlined), the residues surrounding the active-site cleft (wave underlined), and the residues surrounding the disulfide bond (dotted underlined) (Tables 1 and 2). Similar to MtGpx0, the surrounding environment of the active-site cleft in bacterial Gpx-like proteins is a mixture of non-charged and charged residues (Table 2). More importantly, most bacterial Gpx-like proteins also lack the dimerization domain and the oligomerization domain, indicating their monomeric state with the exception of a small proportion of proteins (17%) containing the oligomerization loop (Fig. S4). The four domains also harbor the conserved residues located on the core elements of the tertiary structure, such as the aliphatic group residues Gly and Pro, residing in the turn/bend of the tertiary structures, and the hydrophobic group residues Phe, Trp, Val, Leu, and Ile, constituting the inner β-strands of the tertiary structure (Table 3). Those conserved residues in the four domains are the key elements forming the inner core of the thioredoxin-fold (Fig. S4C).

Taken together, the Gpx-like proteins from isolated bacterial species, similar to those from environmental microbiome, plants and fungi, share common structural elements and functional motifs specific for TRX-dependent catalysis.

### Phylogenetic Structure of Bacterial Gpx or Gpx-Like Proteins

To investigate the sequence diversity and phylogenetic structures of the bacterial Gpx-like proteins, we created a non-redundant protein set by merging the protein sequences with similarities greater than 70%, thus obtaining 376 representative sequences for phylogeny construction. Interestingly, the phylogeny exhibits a star-like structure and most of the branches have a bootstrap support value lower than 70, with the exception of some external branches (collapsed as triangles in Fig. S6A). The low bootstrap confidence in internal branches is further illustrated by the highly inter-connected phylogenetic network constructed by SplitsTree (Fig. S6B) [33].

Another notable feature of the phylogeny is that the proteins from the same phylum scatter across multiple branches while the single branches contain a mixture of proteins from multiple phyla. This highly intermingled phylogeny of bacterial Gpx-like proteins is in contrast with the clear grouping structure of canonical Gpx proteins from vertebrates, where the Gpx proteins were clustered in several clades according to their substrates or subcellular localizations [18]. The phylogenetic differences of Gpx proteins between bacteria and vertebrates further support the view that the TRX-dependent, Gpx-like proteins in bacteria are very ancient and have evolved for a long time from a common ancestor, while the occurrence of GSH-dependent, canonical Gpx proteins from vertebrates might be a recent event [17].

## Discussion

Peroxiredoxins (Prx) and glutathione peroxidases (Gpx), usually using TRX and GSH as reducing agents, respectively, have attracted extensive research interest due to their primary roles in protecting cells from oxidative damage. The rapidly accumulated research findings revealed the surprisingly high versatility and complexity of these two families of proteins regarding their catalytic mechanisms, sequence classifications, and evolution. In addition to the heterogeneous subtypes with subtle differences within each family, the two families of proteins also exhibit interconnections with respect to function and evolution. Previous studies have identified multiple Gpx-like peroxidases in diverse organisms, including fungi [8, 9], plant [10, 11], insect [12], and rodent parasites [13-15], in which the Gpx-like proteins exhibit significant sequence homologies with canonical Gpx proteins but higher substrate specificity for TRX than GSH. It has been proposed that most of the non-mammalian glutathione peroxidases should be classified as Gpx-like peroxiredoxins with TRX as the reducing agent [18]. However, the properties and classification of this family of proteins in prokaryotes, particularly in the environmental microbiome, have not been systematically studied.

In this study, we addressed these issues for the first time by comprehensively analyzing the Gpx-like proteins from environmental microbiome metagenomes using public databases and our own dataset [21]. We also analyzed the Gpx-like proteins from individually isolated bacteria by mining a set of about 2,000 publicly available sequences, which is thus far the largest dataset in this regard. By performing primary sequence and tertiary structural analysis, we show that the Gpx-like proteins from the environmental microbiome and isolated bacteria share high sequence homologies with the canonical Gpx proteins but use TRX as the reductant and should therefore be characterized as TRX-dependent peroxiredoxins. This is the following evidence: (i) the conservation of the peroxidatic Cys in place of SeCys; (ii) the existence of a second “resolving Cys” in the helix α1a for forming the disulfide bond with the peroxidatic Cys through conformational change; and (iii) the absence of the dimerization domains and oligomerization domain required for the formation of multimeric structure. These three features were proposed as the minimal requisites for classifying Gpx-like proteins as TRX-dependent peroxiredoxins [17].

Of particular interest, a small proportion of sequences from the environmental microbiome (4.2%) and isolated bacteria species (17%) contain the oligomerization loop domain but lack the dimerization loop domain (Figs. S1 and S4). Based on parsimony evolution, it is reasonable to propose that the occurrence of the oligomerization loop is a late evolutionary event that succeeds the ancestral sequences not containing the loop. However, this oligomerization loop was not observed in Gpx-like proteins from fungi, insects or higher plants based on the analysis of the data from PeroxiBase [2]. It appears that the small proportion of bacterial sequences containing this loop are the only Gpx-like proteins other than the canonical mammalian Gpx, which “evolved” this loop. This loop is a manifestation of the interconnection between the two groups of proteins, Prx and Gpx. Moreover, based on the previous consensus that the bacterial Gpx-like proteins are more ancient than the canonical mammalian Gpx proteins [17], we propose two possible explanations for the sequences containing the oligomerization loop: (i) They are the remnants of GSH-dependent survivors in bacteria in a GSH-deficient and TRX-rich environment, but were later adopted by mammals facing a GSH-rich condition. (ii) They could be a consequence of convergent evolution between some bacteria in a GSH-only environment and mammals in the normal GSH-rich condition.

Regardless, the external environments played key roles in shaping the evolution of the two groups of proteins. The TRX-dependent catalysis of Gpx-like proteins in bacteria and GSH-dependent catalysis of Gpx in mammals might simply reflect the availability of TRX or GSH in their cellular environments. In bacteria, GSH is very limited or absent, especially in gram-positive bacteria [39]. Therefore, bacteria still maintain the Cys-based Gpx using the highly available TRX as the reducing agent. The mammalian Gpx proteins may have evolved due to the high concentration of GSH in eukaryotic cells and the higher catalytic efficiency of the SeCys than Cys. It revealed an evolutionary achievement in the shift of reducing agents by only mutating a small amount of key elements without drastically changing sequences.

The shaping effects of the environments were also manifested by the phylogenetic structure of the Gpx-like proteins of bacteria. The availability of the large amount of Gpx sequences allowed us to build a phylogeny capturing a sufficient diversity of bacterial taxonomy. The phylogeny of the bacterial Gpx-like proteins exhibits a star-like radiating structure, where the proteins are unable to form confident branch splitting. This structure is different from the previous report in [18], where the bacterial Gpx-like proteins form three distinct groups. It is probably due to the small amount of sequences (fewer than 50) included in the study of [18]. The star-like structure is also in clear contrast to the situation for the canonical Gpx proteins in mammals [18], where the Gpx proteins were clustered in several well-defined clades. In comparison with the relatively narrow living environments of mammals, bacteria inhabit a wide spectrum of environmental conditions and thus may face diverse oxidative challenges in these environments. The highly diversified genetic profile of Gpx-like proteins in bacteria might be the consequence of adapting to distinct oxidative stresses encountered in a wide range of environmental niches.

In conclusion, we have comprehensively analyzed the Gpx-like proteins encoded by the environmental microbiome and isolated bacterial species, and identified the characteristics of the proteins in terms of sequences, structures, and phylogeny. We showed that the Gpx-like proteins from bacteria should be classified as TRX-dependent peroxiredoxins using TRX as the reductant rather than GSH. The high diversity of this group of proteins may provide a genetic pool to be used as a resource for antioxidant applications or targets of antibacterial agents. In the meantime, the huge diversity of the proteins poses challenges for future functional and application studies. Novel methods of high-throughput screening are needed to identify the sequences with high antioxidant capability under specific oxidative stresses. Also, the physiological relationship between the Gpx-like proteins and the Prx proteins, and the regulatory network within bacteria, remain to be delineated and that will help to enhance the understanding of the molecular mechanisms of bacteria in protecting cells from diverse oxidative damage.

## Supplemental Materials

Supplementary data for this paper are available on-line only at http://jmb.or.kr.

## Figures and Tables

**Table 1:** Sequence patterns of the four conserved domains in bacterial Gpx-like proteins.

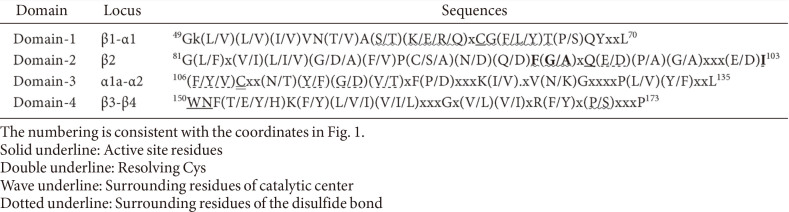

**Table 2:** Conserved residues in the catalytic center in bacterial Gpx-like proteins (consensus proportion ≥ 70%).

Consensus	Conservation	Residue types	Structure/Function
Active site residues			
Cys61	100%	Cys	catalytic residue, perioxidatic Cys
Gln95	95%	Gln	catalytic residue
Trp150	100%	Trp	catalytic residue
Asn151	100%	Asn	catalytic residue
Cys107	94%	Cys	resolving Cys
Surrounding residues of catalytic center			
Ser59	85%	Ser/Thr	turn, surrounding the catalytic center
Lys60	49%	Lys/Glu/Arg/Gln	charged residues in the surrounding of the catalytic center
Gly62	96%	Gly	turn, surrounding of the catalytic center
Phe63	50%	Phe/Leu/Tyr	hydrophobic loop, surrounding the catalytic center
Thr64	89%	Thr/Ala/Ser	hydrophobic loop, surrounding the catalytic center
Phe92	100%	Phe	turn, hydrophobic loop, surrounding the catalytic center
Gly93	41%	Gly/Ala	turn, hydrophobic loop, surrounding the catalytic center
Pro169	71%	Pro/Ser	turn, surrounding the catalytic center
Surrounding residues of the disulfide bond			
Glu96	82%	Glu/Asp	loop connecting β2 and α1a
Ile103	83%	Ile	buried hydrophobic
Phe106	73%	Phe/Tyr/Val	buried hydrophobic
Tyr111	72%	Try/Phe	buried hydrophobic
Gly112	78%	Gly/Asp	turn
Val113	87%	Val/Thr	hydrophobic

**Table 3:** Conserved residues in basic structure elements in bacterial Gpx proteins (consensus proportion ≥ 70%).

Consensus	Conservation	Residue types	Location/Structure
Residues in the inner core of the structure			
Leu53	88%	Leu/Val	buried hydrophobic on β1
Val55	98%	Val	buried hydrophobic on β1
Asn56	100%	Asn	β1
Ala58	100%	Ala	β1
Gln66	100%	Gln	buried hydrophobic on α1
Tyr67	74%	Tyr/Phe/Leu	buried hydrophobic on α1
Leu70	100%	Leu	buried hydrophobic on α1
Leu73	80%	Leu/Ile/Val	buried hydrophobic α1
Tyr77	72%	Tyr/Phe/Trp/Leu	α1
Gly81	90%	Gly/Asn/Asp	loop connecting α1 and β2
Leu85	81%	Leu/Ile/Val	buried hydrophobic on β2
Gly86	81%	Gly/Asp/Ala/Ser	buried hydrophobic on β2
Phe87	79%	Phe/Val/Leu	buried hydrophobic on β2
Asn90	88%	Asn/Asp	polar turn, connecting β2 and α1a
Gln91	70%	Gln/Asp/Asn/Glu	polar turn, connecting β2 and α1a
Lys120	88%	Lys/Pro/Arg/Thr	end of β2a
Leu131	86%	Leu/Val/Ile/Phe	α2
Tyr132	61%	Tyr/Phe/Trp	buried hydrophobic on α2
Leu135	92%	Leu/Met	buried hydrophobic on α2
Phe152	100%	Phe	turn preceding β3
Lys154	100%	Lys	buried hydrophobic and charged residue on β3
Phe155	83%	Phe/Tyr	buried hydrophobic on β3
Leu156	85%	Leu/Val/Ile	buried hydrophobic on β3
Val163	75%	Val/Leu/Pro/Ile	β4
Arg166	82%	Arg/Ser/Gln	buried hydrophobic on β4
Phe167	70%	Phe/Tyr	β4
Ile180	78%	Ile/Val/Leu	buried hydrophobic on α3
Leu184	87%	Leu/Ile/Val	buried hydrophobic on α3
Residues at the turn/bend			
Gly49	88%	Gly/Asp/Asn	turn preceding β1
Pro88	100%	Pro	turn, end of β2
Pro97	83%	Pro/Ala/Leu/Lys/Ser	bend preceding α1a
Gly98	83%	Gly/Ala/Asp/Glu/Lys	bend preceding α1a
Phe115	100%	Phe	bend preceding β2a
Pro116	84%	Pro/Thr/Gln	turn at the start of β2a
Val123	100%	Val	bend between β2a and α2
Gly125	100%	Gly	bend between β2a and α2
Pro130	91%	Pro/Ser	turn at the start of α2
Gly161	98%	Gly	bend preceding β4
Pro173	94%	Pro/Ala	turn at the start of α3
