# Supplementary material for: Analysis and Characterization of Glutathione Peroxidases in an Environmental Microbiome and Isolated Bacterial Microorganisms
Source: J Microbiol Biotechnol. 2023 Jan 20;33(3):299–309. doi: 10.4014/jmb.2209.09006 (PMC10084747; doi:10.4014/jmb.2209.09006)
Supplement: Supplementary file 1 [file jmb-33-3-299-supple.pdf]

J. Microbiol. Biotechnol.
https://doi.org/10.4014/jmb.2209.09006

Fig.S1

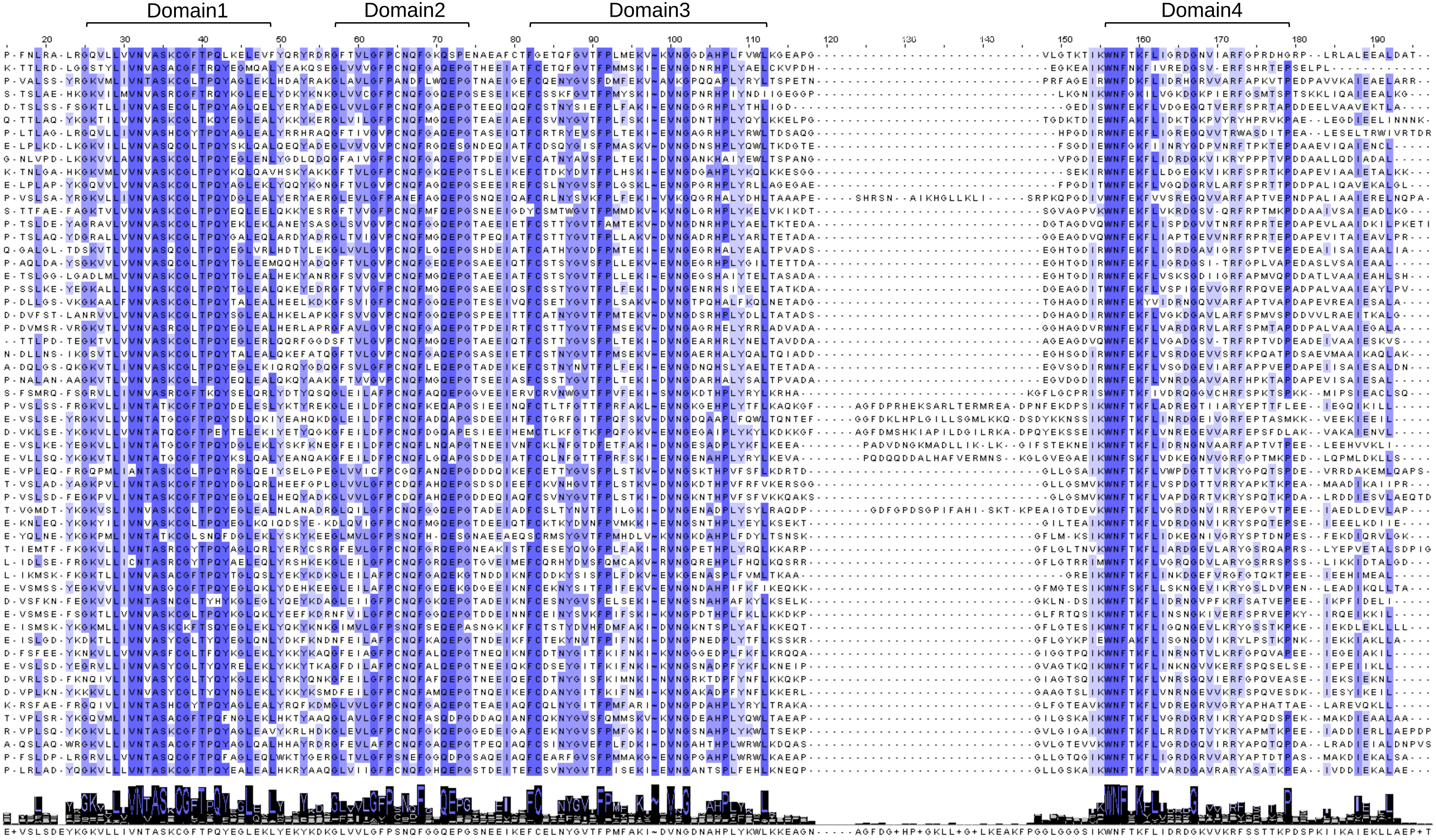

Fig. S1: Profiling of conservation patterns of Gpx-like proteins from environmental microbiome.

Multiple sequence alignment was performed for 392 non-redundant proteins (pair-wise similarity lower than 80%) and the conservation pattern was profiled and depicted with gradient blue color. The highly divergent N-terminus is not shown.

Fig.S2

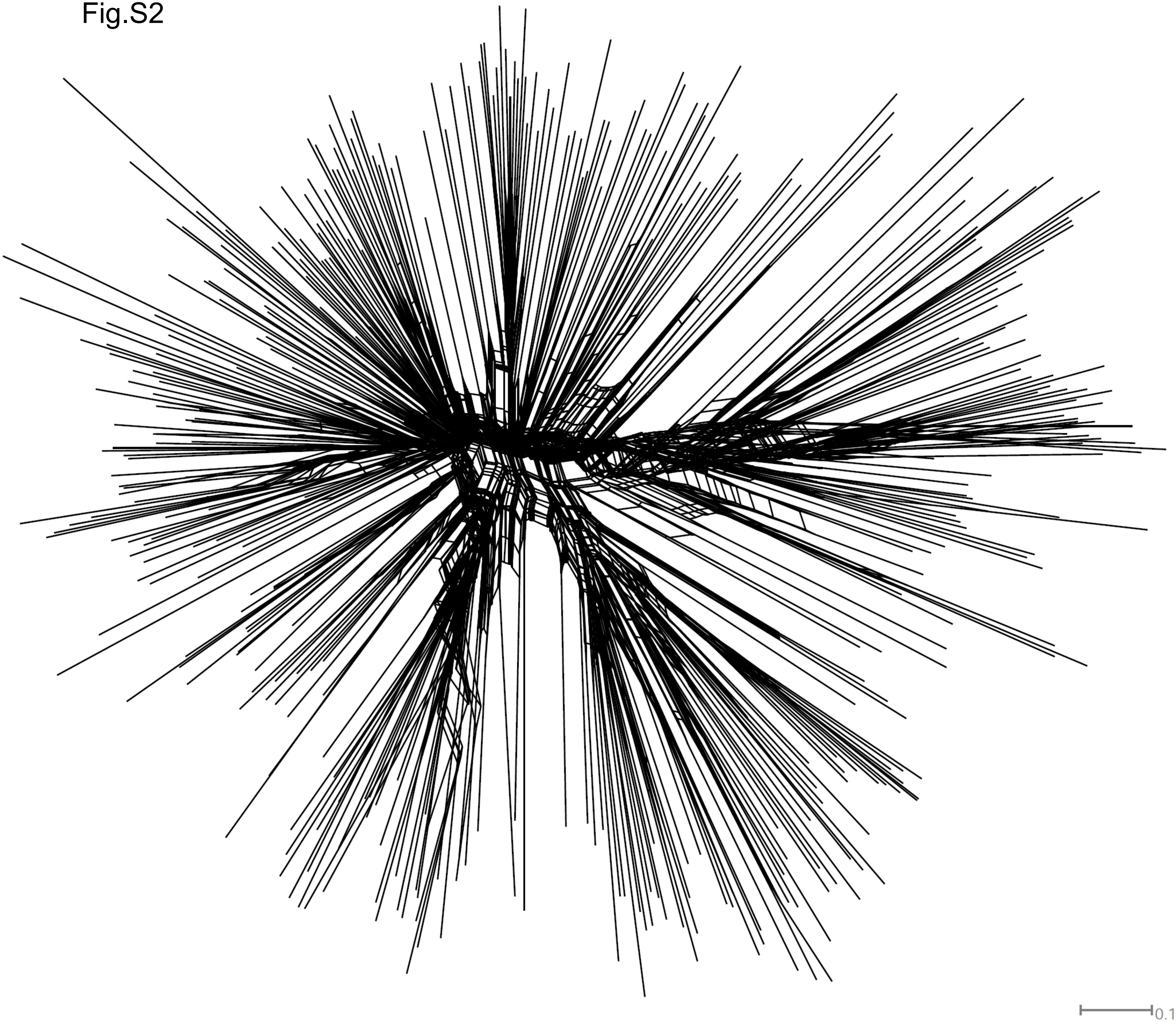

Fig. S2: The phylogenetic network of the 392 non-redundant microbiome-encoded Gpx-like proteins. The phylogeny was built with SplitsTree using the neighbor-net method.

Fig.S3

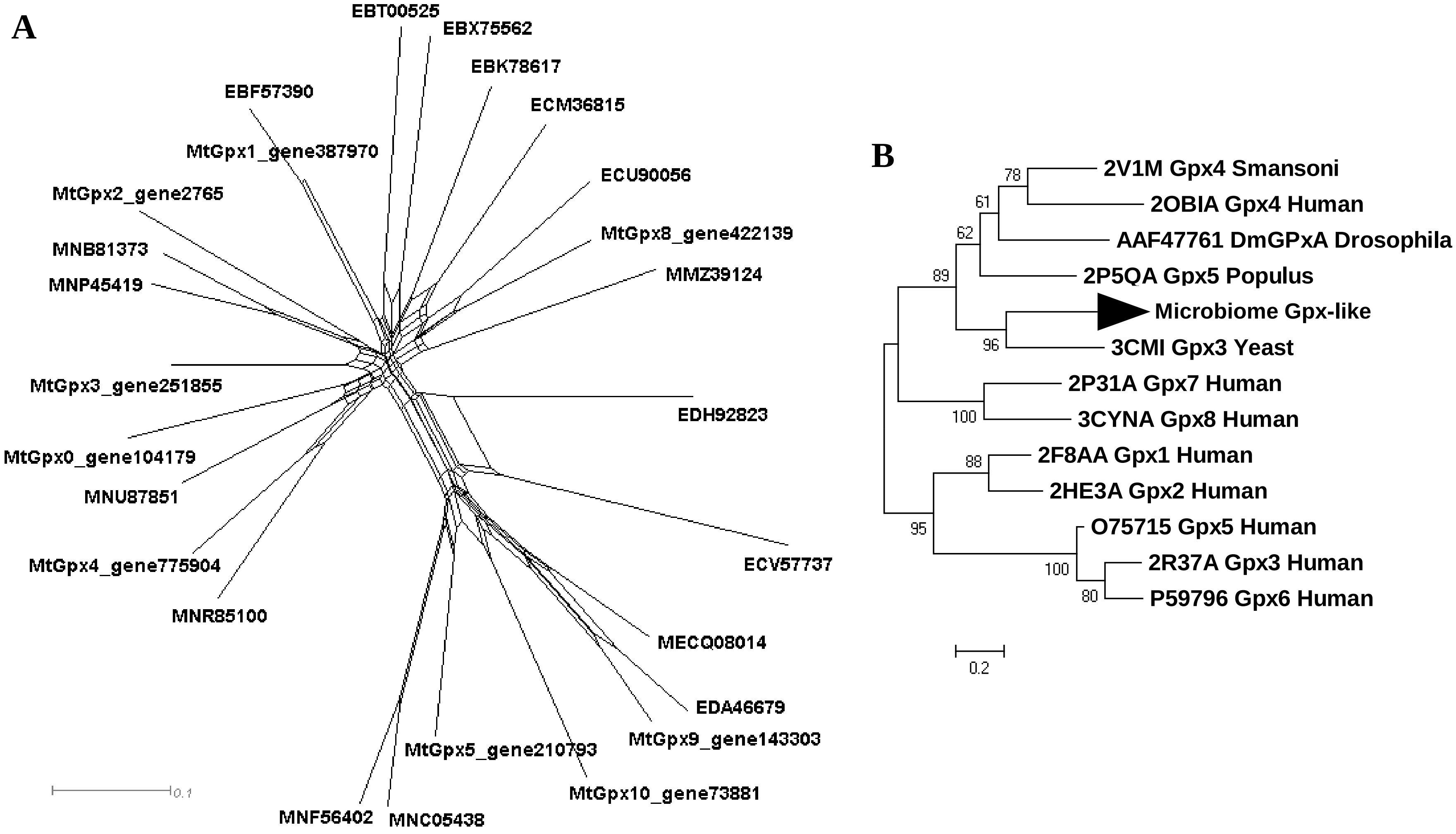
A
EBT00525
EBX75562
EBK78617
EBF57390
ECM36815
MtGpx1_gene387970
ECU90056
MtGpx2_gene2765
MtGpx8_gene422139
MNB81373
MMZ39124
MNP45419
MtGpx3_gene251855
EDH92823
MtGpx0_gene104179
MNU87851
MtGpx4_gene775904
ECV57737
MNR85100
MECQ08014
EDA46679
MtGpx9_gene143303
MtGpx5_gene210793
MtGpx10_gene73881
MNF56402
MNC05438
0.1
B
78
2V1M Gpx4 Smansoni
61
2OBIA Gpx4 Human
62
AAF47761 DmGPxA Drosophila
89
2P5QA Gpx5 Populus
Microbiome Gpx-like
96
3CMI Gpx3 Yeast
2P31A Gpx7 Human
100
3CYNA Gpx8 Human
88
2F8AA Gpx1 Human
2HE3A Gpx2 Human
95
O75715 Gpx5 Human
100
2R37A Gpx3 Human
80
P59796 Gpx6 Human
0.2

Fig. S3. The phylogeny of the microbiome Gpx-like proteins and those with known structural information. (A) Phylogenetic network of nine representative microbiome-encoded Gpx-like proteins. The network was built with SplitsTree using the neighbor-net method. (B) Phylogenetic tree of microbiome-encoded Gpx-like proteins and those with known structural information from other organisms. The tree was built using neighbor-joining method with bootstrap of 1000.

Fig. S4

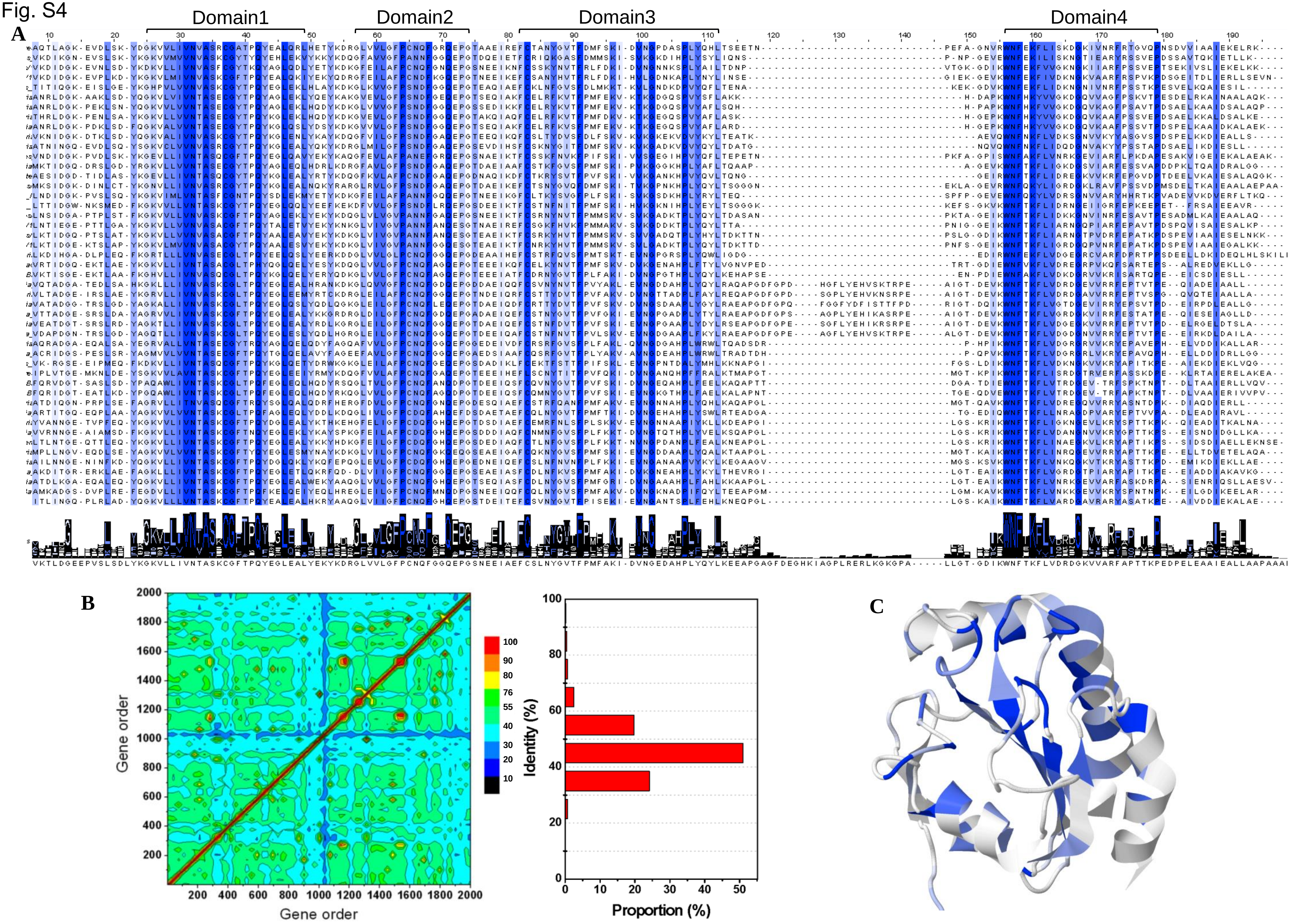

A
Domain1
Domain2
Domain3
Domain4
B
Gene order
Gene order
100
90
80
76
55
40
30
20
10
Identity (%)
Proportion (%)
C

Fig. S4: Profiling of conservation patterns of bacterial Gpx-like proteins. (A) Primary structure alignment was performed for 1997 UniProt entries and the conservation pattern was profiled and depicted with gradient blue color. The highly diverged N-terminus is not shown. A full presentation of the profiling for the 1997 proteins is shown in Fig. S5. (B) Left panel: a heatmap presentation of the pair-wise similarities of the 1997 bacterial Gpx-like proteins. Right panel: histogram distribution of the pair-wise similarities ranging from 35-55% with an average of 45%. (C) Color mapping of the conservation patterns of the aligned Gpx-like protein to the tertiary structure of MtGpx0.

Fig.S5

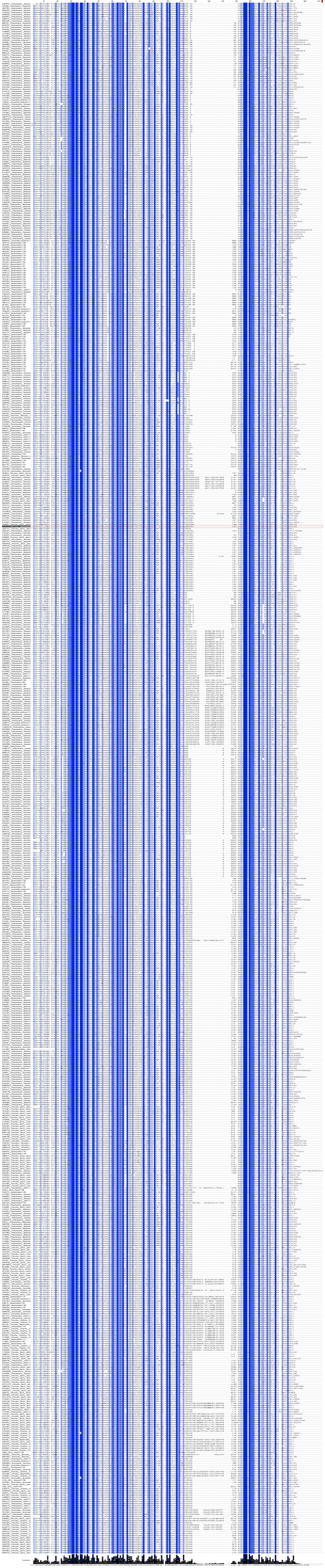

Fig. S5: Multiple sequence alignment and conservation patterns for 1997 bacterial Gpx-like proteins. The conservation pattern is depicted with gradient blue color. The conservation logo is shown at the bottom of the alignment.

Fig.S6

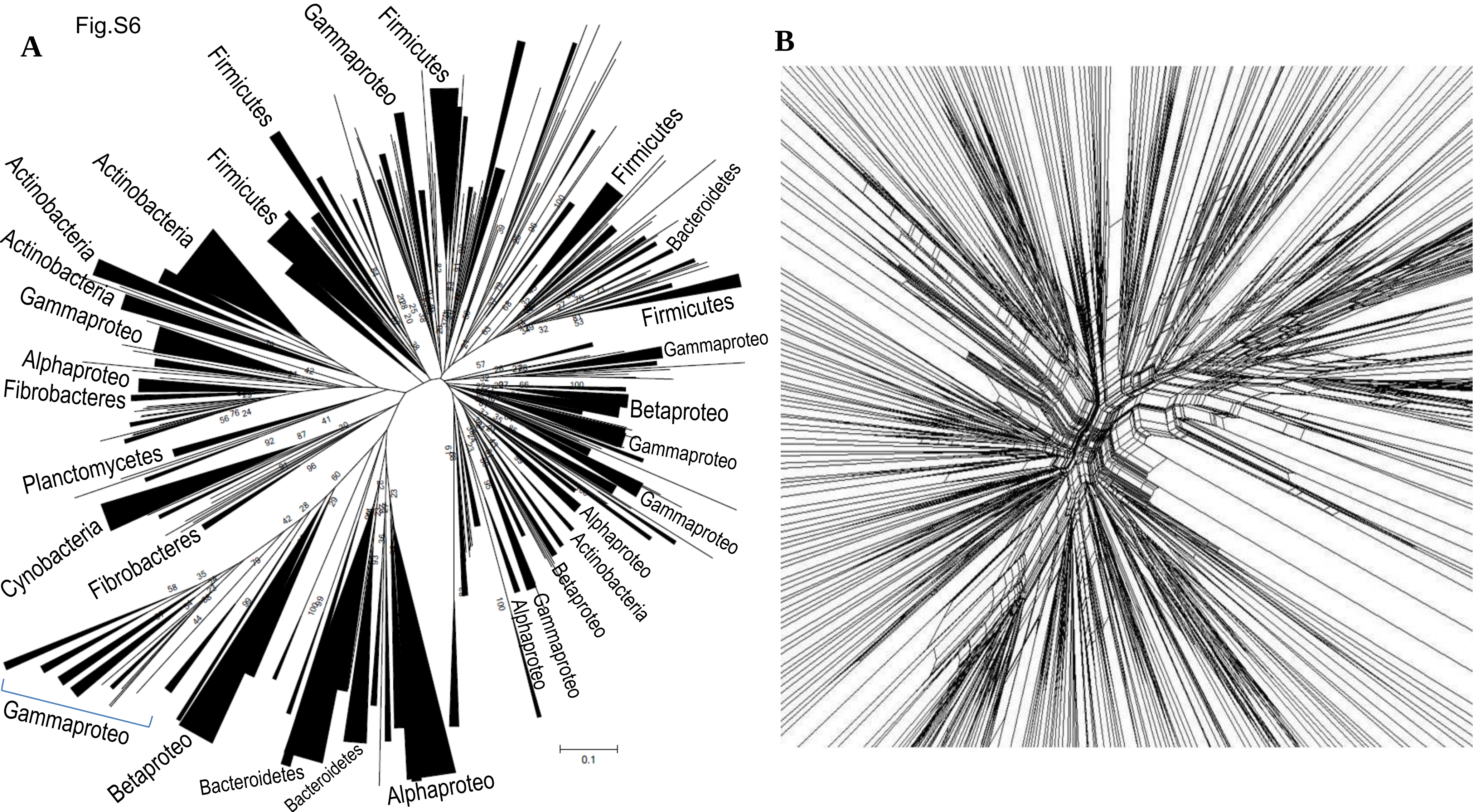
A
B
Firmicutes
Gammaproteo
Firmicutes
Actinobacteria
Actinobacteria
Firmicutes
Actinobacteria
Gammaproteo
Alphaproteo
Fibrobacteres
Planctomycetes
Cynobacteria
Fibrobacteres
Gammaproteo
Betaproteo
Bacteroidetes
Bacteroidetes
Alphaproteo
Alphaproteo
Gammaproteo
Betaproteo
Actinobacteria
Alphaproteo
Gammaproteo
Gammaproteo
Betaproteo
Gammaproteo
Firmicutes
Bacteroidetes
Firmicutes
0.1

Fig. S6: Phylogenetic structure of the non-redundant set of the 376 bacterial Gpx-like proteins. (A) The tree was built with the neighbor-joining method with bootstrap of 2000 using MEGA6. The tree does not exhibit confident grouping except for several external branches, but exhibits a star-like structure. (B) The phylogenetic network was also built for the same set of bacterial Gpx-like sequences using SplitsTree with the neighbor-net method. The phylogeny is highly interconnected.
